# Diminished responses to mRNA-based SARS-CoV-2 vaccines in individuals with rheumatoid arthritis on immune-modifying therapies

**DOI:** 10.1172/jci.insight.168663

**Published:** 2023-08-08

**Authors:** Samuel D. Klebanoff, Lauren B. Rodda, Chihiro Morishima, Mark H. Wener, Yevgeniy Yuzefpolskiy, Estelle Bettelli, Jane H. Buckner, Cate Speake, Marion Pepper, Daniel J. Campbell

**Affiliations:** 1 Benaroya Research Institute, Seattle, Washington, USA.; 2 Department of Immunology and; 3 Department of Laboratory Medicine and Pathology, University of Washington School of Medicine, Seattle, Washington, USA.

**Keywords:** Autoimmunity, Vaccines, Adaptive immunity, Costimulation

## Abstract

Rheumatoid arthritis (RA) is a chronic inflammatory autoimmune disorder that causes debilitating swelling and destruction of the joints. People with RA are treated with drugs that actively suppress one or more parts of their immune system, and these may alter the response to vaccination against SARS-CoV-2. In this study, we analyzed blood samples from a cohort of patients with RA after receiving a 2-dose mRNA COVID-19 vaccine regimen. Our data show that individuals on the cytotoxic T lymphocyte antigen 4–Ig therapy abatacept had reduced levels of SARS-CoV-2–neutralizing antibodies after vaccination. At the cellular level, these patients showed reduced activation and class switching of SARS-CoV-2–specific B cells, as well as reduced numbers and impaired helper cytokine production by SARS-CoV-2–specific CD4^+^ T cells. Individuals on methotrexate showed similar but less severe defects in vaccine response, whereas individuals on the B cell–depleting therapy rituximab had a near-total loss of antibody production after vaccination. These data define a specific cellular phenotype associated with impaired response to SARS-CoV-2 vaccination in patients with RA on different immune-modifying therapies and help inform efforts to improve vaccination strategies in this vulnerable population.

## Graphical abstract

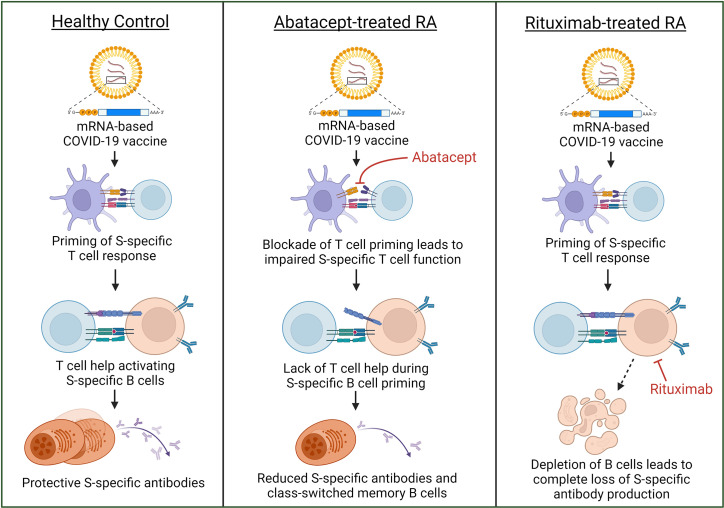

## Introduction

The COVID-19 pandemic, caused by SARS-CoV-2, has resulted in over 6 million deaths and worldwide economic and social disruption. Vaccines targeting the SARS-CoV-2 spike (S) protein are essential tools in combating this pandemic and have proved highly efficacious in preventing severe disease, hospitalization, and death. In the United States, the 2 most common SARS-CoV-2 vaccines are Pfizer’s BNT162b2 and Moderna’s mRNA-1273 vaccines, which use modified mRNA platforms that induce potent cellular and humoral responses to the S protein (1, 2). However, for patients with a compromised immune system, such as those with autoimmune disease taking immunosuppressive therapies, vaccination can often be less effective (3). Although both vaccines showed approximately 95% efficacy at preventing COVID-19 in initial clinical trials, immunocompromised patients were excluded from those trials (4), and a better understanding of the response to COVID-19 vaccination in this patient population is urgently needed. This is especially true given the emergence of viral variants that partially evade antibody-mediated protective immunity because of structural mutations in the S protein.

The response to SARS-CoV-2 mRNA vaccines is characterized by rapid production of S protein–specific antibodies, initially from short-lived plasmablasts and later from a smaller pool of long-lived plasma cells (5, 6). The majority of vaccine-induced neutralizing antibodies target the S protein receptor binding domain (RBD) and contribute to protection by preventing interaction with the angiotensin-converting enzyme 2 (ACE2) receptor on human epithelial cells, thus blocking infection. Serum levels of anti-S antibodies decline slowly over several months but rebound quickly upon administration of subsequent booster vaccine doses or reinfection as S-specific memory B cells generated by the initial vaccination rapidly activate and differentiate into antibody-secreting plasmablasts (5). Vaccination also induces strong CD4^+^ and CD8^+^ T cell responses, as measured by expression of activation markers such as CD69 and CD137 by these cells after stimulation with S protein peptides. Among CD4^+^ T cells, effector and memory T cells producing key antiviral cytokines such as IL-2, IFN-γ, and IL-21 dominate the response, and an expanded population of S-specific T cells persists for at least several months after vaccination (5, 7).

Patients with autoimmune diseases such as rheumatoid arthritis (RA) are treated with drugs that target key immune pathways important for disease pathology but that can impair effective vaccine responses. Indeed, although the American College of Rheumatology has recognized the potential of these therapies to impact SARS-CoV-2 vaccination, there is limited consensus on whether to recommend brief cessation of treatment for patients with RA receiving the SARS-CoV-2 vaccines (8). Conventional disease-modifying antirheumatic drugs are antiinflammatory and immunosuppressive small molecule drugs, the most common of which is methotrexate (MTX), which has become the standard of care for RA. The mechanism of action of MTX in RA has not been fully defined, although it is thought to act via adenosine signaling and blocking folate metabolism in disease-causing lymphocytes (9, 10). Patients whose disease is difficult to control with MTX and other first-line treatments are also treated with recombinant biologic drugs, among which is the cytotoxic T lymphocyte antigen 4–Ig therapy abatacept. Abatacept functions by binding to CD80 and CD86 on antigen-presenting cells, effectively blocking their ability to provide costimulation to pathogenic autoreactive T cells. We and others demonstrated that abatacept treatment reduces the number and activity of circulating T follicular helper (Tfh) cells (11–13), a specialized CD4^+^ T cell population that produces IL-21 and provides help to promote the proliferation, isotype class switching, and affinity maturation of antigen-specific B cells (14). Indeed, costimulation blockade via abatacept inhibits vaccine-induced antibody responses, including to SARS-CoV-2 mRNA vaccines (15–17). This decreased response has been demonstrated both 2 weeks and 6 months after SARS-CoV-2 vaccination (18, 19). Even after a booster dose of SARS-CoV-2 vaccine, abatacept-treated RA patients have reduced antibody responses and reduced memory T and B cell functionality (20). Rituximab, an anti-CD20 antibody that depletes B cells, is also used to treat RA, and as expected, individuals treated with rituximab have severely blunted vaccine responses, including to SARS-CoV-2 mRNA vaccines (21, 22). Indeed, individuals with rheumatic disease on many different immunomodulators, especially rituximab and abatacept, have significantly increased risk of COVID-19 breakthrough infection after vaccination (23), and anti-CD20 therapy also increases the risk of COVID-19–related lethality in patients with multiple sclerosis (MS) (24).

A detailed cellular analysis of T and B cell responses to SARS-CoV-2 vaccination in patients with RA on different immunomodulatory therapies is still lacking. For this study, we assembled a cohort of individuals with RA who were treated with MTX, abatacept, or rituximab and compared their responses to SARS-CoV-2 vaccination with those of healthy control patients. We measured S protein–specific antibody responses in the serum and assessed the abundance, phenotype, and function of SARS-CoV-2–specific T cells and B cells. We found that all cohorts of individuals with RA had altered vaccine responses compared with healthy controls. As expected, the lack of B cells resulted in a near-total loss of anti–SARS-CoV-2 antibodies in rituximab-treated individuals. Abatacept treatment also led to reduced S-specific and neutralizing antibodies. Interestingly, the number of RBD-specific B cells found in peripheral blood was similar in control, abatacept-treated, and MTX-treated patients. However, abatacept reduced B cell class switching to IgG and altered memory B cell differentiation. The number of SARS-CoV-2–specific CD4^+^ T cells was decreased in MTX- and abatacept-treated patients, and production of the key cytokines IL-2, IFN-γ, and IL-21 was also diminished by abatacept treatment. Thus, abatacept treatment limits the efficacy of SARS-CoV-2 mRNA vaccines in a manner consistent with impaired generation of optimal T cell responses capable of providing help to B cells for production of high-titer, class-switched, virus-neutralizing antibodies. Understanding the mechanistic basis for these impaired responses sheds light on the cellular networks required for immune protection in SARS-CoV-2–vaccinated individuals. These results also provide additional support for temporary cessation of abatacept treatment before vaccination when clinically manageable to help ensure optimal vaccine-induced immune protection from SARS-CoV-2 infection.

## Results

### Abatacept and rituximab reduce humoral immune responses to SARS-CoV-2 vaccination.

**Figure 1:**
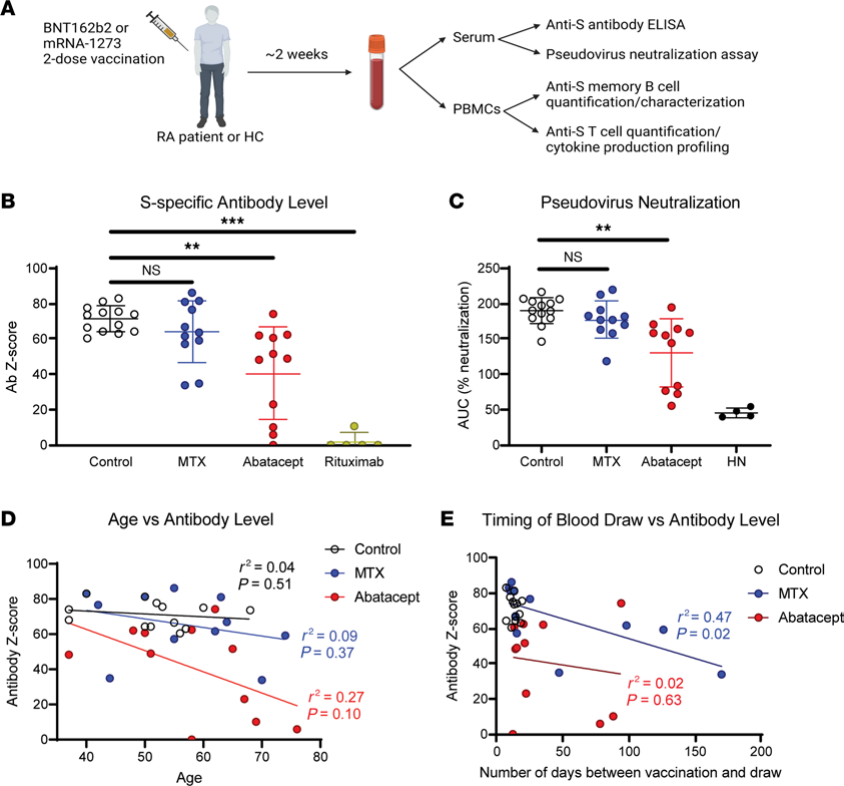
Abatacept and rituximab reduce SARS-CoV-2–specific antibody levels after vaccination. (**A**) Study schematic. (**B**) Normalized anti-S antibody levels as measured by ELISA. (**C**) Pseudovirus neutralization of patients’ sera, as AUC across serum dilutions, with historical/naive (HN) control. (**D**) Patient age graphed against anti-S antibody levels. (**E**) Time between each patient’s second vaccine dose and blood draw for the study graphed against anti-S antibody levels. Error bars represent mean ± SD. Linear regression shown with *r*^2^ values and *P* values testing probability of a nonzero slope. Statistics determined by Kruskal-Wallis test with post hoc Dunn’s multiple-comparison test. **P < 0.01, ***P < 0.001.

Our study cohort consisted of 40 individuals, including 13 healthy controls and 27 patients with RA (Supplemental Table 1; supplemental material available online with this article; https://doi.org/10.1172/jci.insight.168663DS1). Eleven participants with RA were being treated with MTX, 11 were being treated with abatacept (6 of whom were also on additional therapies including hydroxychloroquine, prednisone, leflunomide, and sulfasalazine), and 5 were being treated with rituximab (3 of whom were also on additional therapies including MTX, hydroxychloroquine, and leflunomide). Healthy controls were selected to be approximately age and sex matched to the RA cohort. All study participants donated a single blood sample after receiving the second dose of either the Pfizer BNT162b2 or Moderna mRNA-1273 vaccine. Donors were requested to provide a blood sample within 1–3 weeks after vaccination, but in some cases participants with RA donated blood at their next clinical visit — which generally occurred within 3 months of vaccination. For all blood samples collected, serum and PBMCs were isolated and subjected to humoral and cellular analyses (Figure 1A).

Generation of virus-neutralizing antibodies is the primary goal of vaccination, and they correlate strongly with protection from SARS-CoV-2 (6, 25). Using ELISA to measure S protein–specific IgG and normalizing to a historical negative control group (Figure 1B), we found as expected that patients on the B cell–depleting therapy rituximab showed almost no detectable level of antibodies in their serum, whereas patients on abatacept generated significantly lower levels of S-specific antibodies than healthy controls. We did not observe a significant decrease in the antibody response in MTX-treated patients, although responses trended lower in a subset of these individuals.

In addition to measuring anti-S antibody levels, we conducted a pseudovirus neutralization (pVNT) assay in which lentiviral particles pseudotyped with the SARS-CoV-2 S protein (from the ancestral WA-1 strain used in the vaccine) are incubated with serum to measure blockade of infection of ACE2-expressing target cells. Samples from SARS-CoV-2–naive and unvaccinated individuals drawn in early 2020 were included as historical negative controls, and we used a monoclonal anti-RBD antibody as a positive control (Supplemental Figure 1A). As with the total antibody levels, we found that abatacept, but not MTX, significantly decreased serum neutralization activity compared with healthy controls (Figure 1C). Indeed, we observed a strong correlation between total anti-S antibody IgG levels and pseudovirus neutralization among our patients (Supplemental Figure 1B), indicating that although quantitatively impaired, the quality of antibody produced in MTX- and abatacept-treated patients was largely normal. We also performed a pVNT assay using lentivirus pseudotyped with S protein from the SARS-CoV-2 Omicron BA.1 variant, to measure the cross-reactive neutralization ability of patients’ serum (Supplemental Figure 1C). We observed low neutralization activity against BA.1 in all patients. Although there were no significant differences between treatment groups, neutralization of BA.1 trended lowest in abatacept-treated patients (Supplemental Figure 1D).

Immune function declines with age, and therefore patient age is a potentially confounding variable in our study. In healthy control and MTX-treated patients, anti-S antibody levels showed no discernible correlation with patient age, whereas in abatacept-treated patients there was a slight negative correlation with age that was not statistically significant (Figure 1D). Another potentially confounding variable is the time between completion of the vaccine series and sample acquisition for our study. This variable is particularly important to address since our control samples were all obtained within 3 weeks of vaccination, whereas samples from individuals with RA were collected as late as 6 months postvaccination. However, we did not observe a significant correlation between the time of sample collection and anti-S antibody levels or pseudovirus neutralization activity in either the MTX- or abatacept-treated groups (Figure 1E and Supplemental Figure 1E). Thus, differences in age or sample timing did not account for the diminished antibody production we observed in abatacept-treated patients. Additionally, we found no difference in antibody production between patients on abatacept monotherapy versus patients on abatacept in combination with any other therapy (Supplemental Figure 1F).

### Abatacept reduces activation and class switching of RBD-specific memory B cells in response to SARS-CoV-2 vaccination.

**Figure 2:**
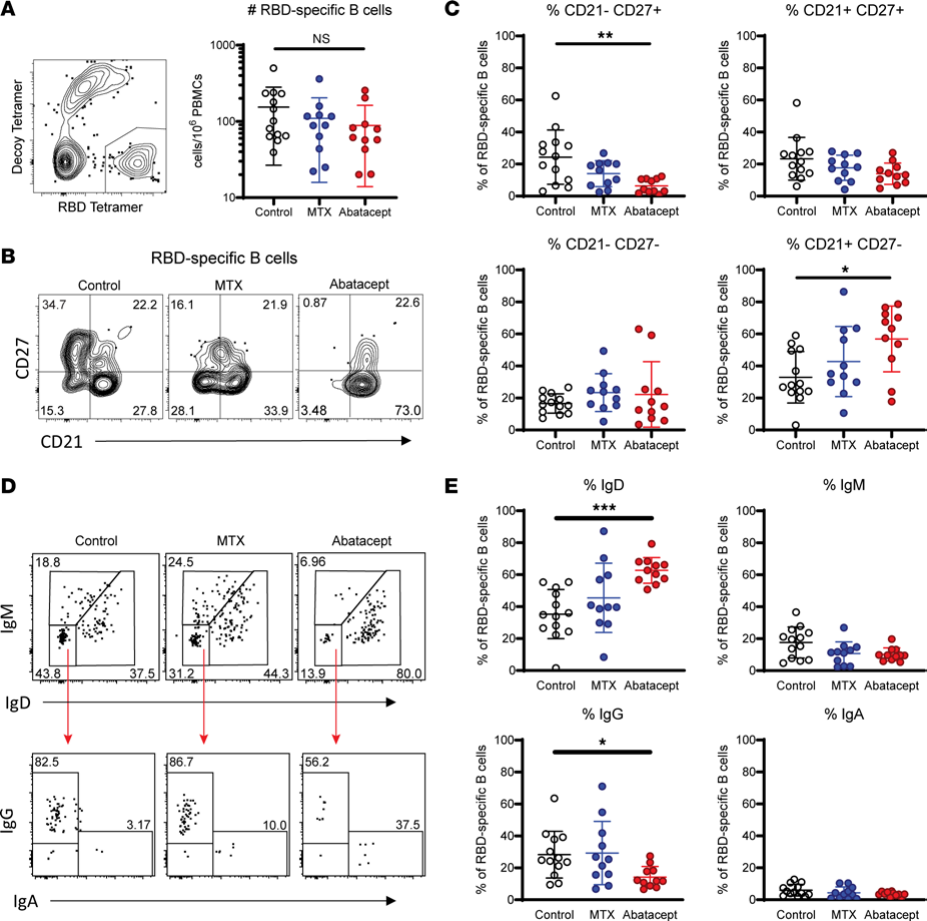
Abatacept treatment reduces activation and class switching in RBD-specific MBCs after vaccination. (**A**) Representative gating on live CD3^–^CD14^–^CD16^–^CD19^+^CD20^+^ B cells (left) and number (right) of SARS-CoV-2 RBD-specific B cells (RBD tetramer^+^decoy tetramer^−^) from PBMCs from control (white), methotrexate-treated (MTX, blue), and abatacept-treated (red) individuals. (**B**) Representative gating on RBD-specific CD38^lo^ nonplasmablast B cells for naive B cells (CD21^+^CD27^−^), classical MBCs (CD21^+^CD27^+^), activated MBCs (CD21^−^CD27^+^), and double-negative activated MBCs (CD21^−^CD27^−^). (**C**) Proportion of RBD-specific B cells that are each phenotype from individuals in the indicated treatment group. (**D**) Representative gating on RBD-specific CD38^lo^ nonplasmablast B cells for isotypes IgD, IgM, IgG, and IgA. (**E**) Proportion of RBD-specific B cells expressing the isotypes indicated in the groups indicated. Data combined from 4 individual experiments. Error bars represent mean ± SD. Statistics determined by Kruskal-Wallis test with post hoc Dunn’s multiple-comparison test. All statistically significant comparisons (*P* < 0.05) are shown. *P < 0.05, **P < 0.01, ***P < 0.001. MBCs, memory B cells.

Diminished antibody responses to SARS-CoV-2 vaccination indicate that the activation and functional differentiation of SARS-CoV-2–specific B cells in abatacept-treated patients may be altered. Therefore, we used RBD tetramer probes to characterize vaccine-induced RBD-specific B cell responses in blood samples from our MTX- and abatacept-treated RA cohorts (25). Although our measurement of serum antibody levels used an ELISA against the entire S protein (not just the RBD), RBD-specific antibodies account for the vast majority of SARS-CoV-2 neutralization (26), and thus, we focused our analyses on B cells that make these key RBD-specific antibodies. We used a decoy tetramer (containing all elements of the tetramer except the RBD) to control for nonspecific binding, then performed magnetic enrichment to increase the frequency of RBD binding among analyzed cells (Figure 2A). Despite diminished antibody responses in patients on abatacept, the total numbers of RBD-specific B cells were statistically similar among all our cohorts (Figure 2A and Supplemental Figure 2A).

After detecting their antigen, CD21^+^CD27^–^ naive B cells proliferate and become CD21^–^CD27^+/–^ activated B cells and then differentiate into resting CD21^+^CD27^+^ classical MBCs, which can rapidly produce protective antibodies upon a reinfection (27). Following vaccination, abatacept treatment was associated with a significantly lower proportion of CD21^–^CD27^+^ RBD-specific B cells and a higher proportion that retained a CD21^+^CD27^–^ naive phenotype compared with healthy control or MTX cohorts (Figure 2, B and C). Since the proportion of activated MBCs declines with time after vaccination (5), we tested the contribution of timing to the depressed RBD-specific B cell activation in the abatacept group by correlating this proportion to the time of blood draw after vaccination. While these factors were negatively correlated for healthy controls (Supplemental Figure 2B), there was no correlation with time after vaccination in either the MTX or abatacept cohorts (Supplemental Figure 2C), suggesting timing does not explain the difference in activation. In contrast, the proportion of classical MBCs (CD21^+^CD27^+^) was not different between controls and treated patients (Figure 2C). Finally, vaccination did not induce significant proportions of CD21^–^CD27^–^CD11c^+^ atypical MBCs, which are associated with aberrant B cell activation in some viral infections (28), in any of our patient groups (Supplemental Figure 2D). Thus, the phenotypic changes we observed are reflected in fewer antigen-experienced activated and memory RBD-specific B cells generated by vaccination in the context of abatacept treatment (Supplemental Figure 2E), although there was not a significant correlation between the number of antigen-experienced MBCs and level of anti-S antibodies in serum from individuals on MTX or abatacept (Supplemental Figure 2F).

The effector function of antibodies depends on their isotype, and the generation of virus-neutralizing IgG is a primary correlate of disease protection in the context of SARS-CoV-2 vaccination (29, 30). Abatacept can interrupt the differentiation and function of Tfh cells that help drive B cell receptor (BCR) class switching from IgD and IgM to predominantly IgG and IgA. Therefore, we assessed the isotype of RBD-specific B cells in each participant group (Figure 2, D and E). Although the MTX cohort showed no significant differences in antibody class switching among RBD-specific B cells compared with controls, abatacept treatment was associated with a significantly lower percentage of IgG^+^ cells and a higher percentage of IgD^+^ cells. This is consistent with the reduced B cell activation and increased proportion of naive phenotype cells in these patients. However, even within the antigen-experienced B cell population, we found abatacept-treated patients had a significantly higher percentage of unswitched IgD^+^ cells and a trend toward a lower percentage of IgG^+^ cells (Supplemental Figure 2G). This indicates that abatacept treatment impairs signals that lead to class switching in addition to those that support naive B cell activation and differentiation into memory.

### MTX and abatacept impair development of S-specific memory T cells after SARS-CoV-2 vaccination.

To determine whether the CD4^+^ T cell response to SARS-CoV-2 vaccination in individuals with RA was impaired compared with controls, we stimulated PBMCs overnight with a pool of peptides from the SARS-CoV-2 S protein, then stained the cells with a T cell activation-induced marker (AIM) flow cytometry panel. We included a negative control vehicle-only stimulation condition (DMSO) and used the commercially available CEFX peptide pool that contains 68 known peptide epitopes from 18 common pathogens that reliably stimulates T cells across a broad range of HLA haplotypes as a positive control (31). We also stimulated cells with a peptide pool from the SARS-CoV-2 membrane/nucleocapsid (M/N) proteins that induces a robust response in individuals previously infected with SARS-CoV-2 but not in vaccinated patients (5).

**Figure 3:**
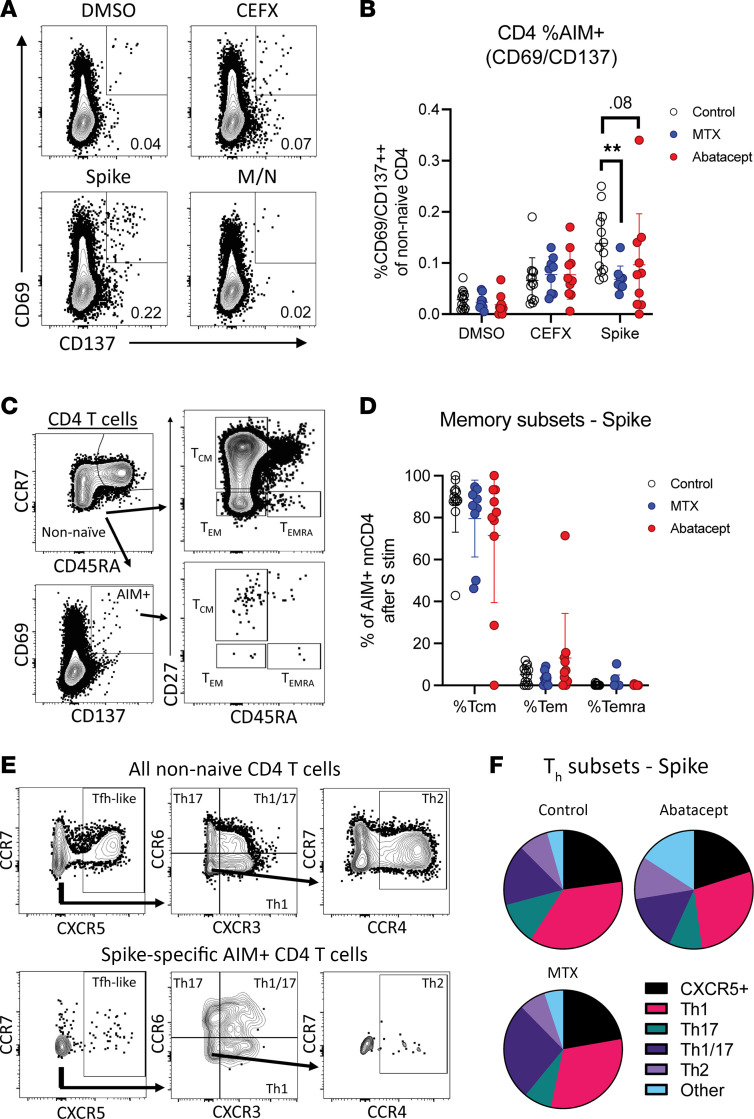
MTX and abatacept reduce S-specific memory T cell responses after vaccination. (**A**) Representative gating of CD3^+^CD45RA^–^CD4^+^ T cells for AIM^+^ (CD69^+^CD137^+^) within indicated stimulation conditions. (**B**) Quantification of AIM expression by patient groups as percentage of CD3^+^CD45RA^–^CD4^+^ cells. (**C**) Representative gating of central memory (CD45RA^–^CD27^+^), effector memory (CD45RA^–^CD27^–^), and Temra (CD45RA^+^CD27^+^) within non-naive and AIM^+^ T cells. (**D**) Quantification of CD4^+^ memory subsets within S protein–stimulated AIM^+^ cells. (**E**) Representative gating of CXCR5^+^ (containing the Tfh population), Th1 (CXCR3^+^CCR6^–^), Th17 (CXCR3^–^CCR6^+^), Th1/17 (CXCR3^+^CCR6^+^), and Th2 (CXCR3^–^CCR6^–^CCR4^+^) cells. (**F**) Pie charts showing percentage of spike-stimulated AIM^+^CD4^+^ T cells falling into each Th subset. Error bars represent mean ± SD. Statistics determined by Kruskal-Wallis test with post hoc Dunn’s multiple-comparison test. All statistically significant comparisons (*P* < 0.05) between treatment groups are shown. **P < 0.01.

We used CD69 and CD137 as representative AIMs, the coexpression of which indicated that a T cell had become activated and was therefore specific for one of the peptides in the stimulation condition (Figure 3A). All groups showed similar CD4^+^ T cell responses to the CEFX pool, and there were no detectible responses to the M/N pool (Supplemental Figure 3A), verifying that the patients in our cohorts were not previously infected with SARS-CoV-2. However, the frequency of vaccine-induced S-specific activated T cells was significantly reduced in MTX-treated patients and also trended lower in the abatacept cohort (Figure 3B). Rituximab-treated patients were also included in these analyses and had lower levels of S-specific AIM^+^ T cells compared with controls (Supplemental Figure 3B). However, due to the small sample size and low numbers of activated cells, this group was excluded from subsequent phenotypic analyses.

We next performed phenotypic characterization of the S-specific T cells, first breaking down the non-naive CD4^+^ T cells into central memory (CD27^+^CD45RA^–^), effector memory (CD27^–^CD45RA^–^), and Temra (CD27^–^CD45RA^+^) subsets (Figure 3C). We observed no significant differences in the proportion of these memory populations in MTX- or abatacept-treated RA participants compared with healthy controls (Figure 3D). We also used differential chemokine receptor expression to identify which functional CD4^+^ T helper subsets were represented in the AIM^+^ cells as previously described (5). In this analysis, we also found no significant difference between groups in the percentage of S-specific CD4^+^ T cells falling into any functional Th subset, and in all cohorts CXCR3^+^ Th1 cells and CXCR3^+^CCR6^+^ Th1/17 cells dominated the response (Figure 3, E and F). However, the phenotypic breakdown of S-specific T cells in individual patients varied widely, and the small numbers of AIM^+^ T cells in many patients reduced our ability to detect significant differences between groups (Supplemental Figure 4).

### Reduced production of Tfh-associated cytokines by S-specific T cells from abatacept-treated patients.

**Figure 4:**
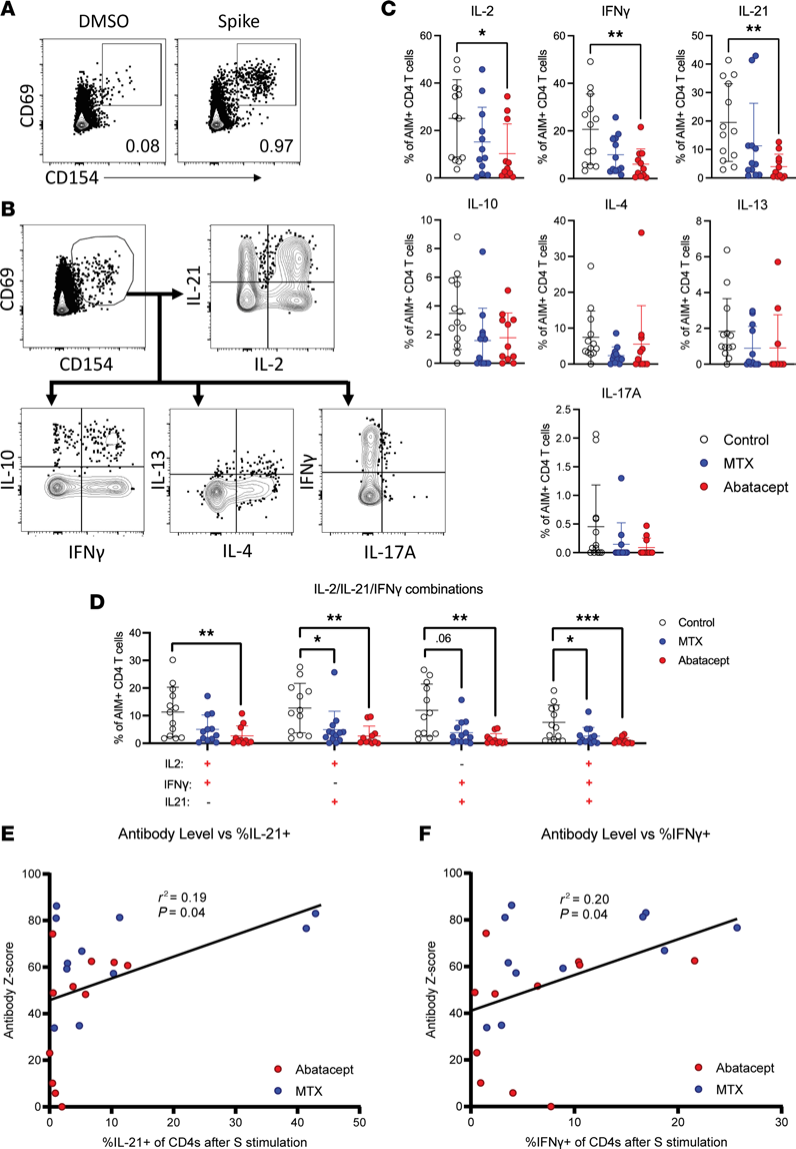
Abatacept-treated patients have reduced Tfh-associated cytokine production by S-specific memory T cells after vaccination. (**A**) Representative gating of AIM^+^ (CD69^+^CD154^+^) T cells for intracellular cytokine staining assay coculture. (**B**) Representative gating of IL-2, IL-21, IL-10, IFN-γ, IL-4, IL-13, and IL-17A expression within AIM^+^CD4^+^ T cells. (**C**) Quantification of the expression of each cytokine by percentage of AIM^+^CD4^+^ T cells. (**D**) Coexpression of IL-2, IL-21, and IFN-γ in each indicated combination. (**E**) Anti-S antibody level graphed percentage of S-activated AIM^+^CD4^+^ T cells expressing IL-21. (**F**) Anti-S antibody level graphed percentage of S-activated AIM^+^CD4^+^ T cells expressing IFN-γ. Error bars represent mean ± SD. Linear regression shown with *r*^2^ values and *P* values testing probability of a nonzero slope. Statistics determined by Kruskal-Wallis test with post hoc Dunn’s multiple-comparison test. All statistically significant comparisons (*P* < 0.05) are shown. *P < 0.05, **P < 0.01, ***P < 0.001.

In addition to the phenotype of S-specific CD4^+^ T cells, we interrogated the ability of these cells to produce antiviral cytokines upon restimulation by performing intracellular cytokine staining on PBMCs stimulated with the S peptide pool. For these experiments, we used CD69 and CD154 as AIMs to identify S protein–specific T cells because of the short stimulation time and our previous observation that CD69^+^CD154^+^ activated cells are the primary cytokine-producing cells when restimulated with S peptide (5, 25) (Figure 4A). Although the markers used to identify activated cells were different, we found that the relative numbers of activated S-specific CD4^+^ T cells in this assay followed the same trend as in our AIM assay using CD69 and CD137 (Supplemental Figure 5).

We analyzed the expression of the cytokines IL-2, IFN-γ, IL-4, IL-13, IL-17A, IL-21, and IL-10 by S-specific CD4^+^ memory T cells from each patient (Figure 4B). As previously reported, the cytokine response to SARS-CoV-2 was heterogenous (5), and we observed substantial fractions of S-specific cells producing each of the analyzed cytokines other than IL-17A. Moreover, we found that the fractions of cells producing IL-2, IFN-γ, or IL-21 were significantly reduced in abatacept-treated patients compared with healthy controls (Figure 4C), and in particular the proportion of cells coexpressing IL-21, IL-2, and IFN-γ was highly reduced in both MTX- and abatacept-treated participants (Figure 4D). IL-21 production is critical for Tfh cell function, and IFN-γ promotes class switching to IgG, suggesting that these T cell defects may be linked to the relatively poor B cell and antibody responses we observed in MTX- and abatacept-treated patients. Indeed, expression of either IL-21 or IFN-γ significantly correlated with S-specific antibody levels in serum of MTX- and abatacept-treated patients (Figure 4, E and F), indicating that these therapies impair humoral immunity by disrupting T cell–B cell collaboration.

## Discussion

Immunosuppressive therapies used for RA can impair responses to vaccination (3). Abatacept and MTX both reduce antibody production in response to various vaccines, including the SARS-CoV-2 mRNA vaccines (15, 16). However, the cellular mechanisms by which these therapies disrupt the complex interactions required for a productive vaccine response are still poorly understood, and the impact this has on vaccine-specific T and B cell memory responses has not been characterized. Here we performed detailed phenotypic and functional characterization of vaccine-elicited T cell and B cell responses in participants with RA treated with different disease-modifying therapies. We present evidence linking specific changes in T and B phenotype to reduced ability to generate anti-S antibodies after vaccination, particularly in the context of the costimulatory blockade therapy abatacept.

As expected, most individuals with RA treated with the B cell–depleting antibody rituximab had undetectable S-specific antibody responses in the serum (32). In addition, the S-specific CD4^+^ T cell responses were substantially reduced compared with controls. This lack of T cell responses in the context of rituximab treatment contrasts with prior studies of MS and B cell–depleting therapies (33, 34), and this could be due to disease-specific effects in RA versus MS, a difference in the specific drugs used in these individuals (rituximab vs. ocrelizumab), the small size of the rituximab cohort in our study (*n* = 5), or the fact that some of the rituximab-treated patients in our study were on additional immunosuppressive drugs, such as hydroxychloroquine or leflunomide.

In MTX-treated patients, we observed a significantly lower magnitude of the S-specific CD4^+^ T cell response to vaccination as measured in our AIM assay. This is consistent with the known mechanism of action of MTX, which inhibits dihydrofolate reductase and thereby attenuates lymphocyte activation and proliferation (35). However, phenotypically and functionally, S-specific CD4^+^ T cells in the context of MTX treatment were similar and not significantly different from those observed in healthy controls. We also observed no significant changes versus healthy controls in the number of RBD-specific B cells, their phenotype, or class switching in our MTX-treated cohort, as well as no significant differences in either the serum S-specific antibody levels or neutralization activity. However, more highly powered studies have detected significant decreases in anti–S protein antibody levels in MTX-treated patients (36, 37), consistent with the altered T cell responses we observed in these patients.

In contrast to the MTX-treated cohort, we found impaired SARS-CoV-2–specific CD4^+^ memory T cell, IgG^+^ memory B cell, and neutralizing antibody responses to SARS-CoV-2 vaccination in abatacept-treated RA participants (38, 39). Although the magnitude of the S-specific CD4^+^ T cell response was not significantly different from that in control patients, we observed a significant reduction in cells producing the key cytokines IL-2, IFN-γ, and IL-21 (20). Abatacept disrupts T cell activation via blockade of CD28-mediated costimulation, and we and others have consistently shown that abatacept treatment is associated with a reduction in Tfh cells (12, 13) and with an impaired transcriptional program of T cell activation and proliferation. Consistent with this, we found that CD4^+^ T cells from the abatacept-treated cohort had reduced levels of Tfh-associated cytokines, particularly IL-21. Abatacept also disrupts Tfh–B cell interactions, which rely on CD28-mediated costimulation (40). Tfh-produced IL-21 and CD154 from these interactions are required for B cell activation and differentiation into germinal center B cells, where they undergo affinity maturation and can differentiate into plasma cells producing high-affinity antibody or MBCs poised to rapidly produce protective antibody upon a reinfection. Germinal center Tfh can produce IL-21, which particularly supports B cell differentiation into plasma cells (41, 42). We also observed decreased production of IFN-γ by S-specific T cells in abatacept-treated patients, and IFN-γ expression normally promotes IgG class switching in B cells. (43). Reduced SARS-CoV-2–specific CD4^+^ T cell IL-21 and IFN-γ production in these patients, and possibly abatacept directly, likely impaired T-dependent activation of SARS-CoV-2–specific B cells, leading to the reduced number of SARS-CoV-2 RBD-specific antigen-experienced B cells, proportion of RBD-specific activated B cells, proportion of RBD-specific IgG^+^ B cells, and neutralizing antibody that we observed.

Neutralizing antibody titers have long been considered an important correlate of protection after vaccination against viral pathogens. Therefore, our finding of reduced anti-S antibodies in the abatacept cohort is clinically relevant for understanding immune protection to SARS-CoV-2 following vaccination in these individuals. Additionally, altered formation of MBCs is also detrimental to immune protection, as these cells are thought to be the primary reservoir of cells responding to SARS-CoV-2 variants that effectively evade vaccine-elicited neutralizing antibody responses. In our prior analyses of abatacept-treated patients, we found that the impact of abatacept on the abundance and transcriptional profile of Tfh was rapidly reversed after drug withdrawal (12). Therefore, when clinically manageable, cessation of abatacept treatment during the course of vaccination is likely to result in significantly improved response to the COVID-19 vaccines, and defense against severe viral infection in the face of future variants.

## Methods

### PBMC and plasma collection.

Participants were enrolled at the Virginia Mason Franciscan Health center, for a vaccine response study through the Benaroya Research Institute. Both healthy controls and patients with RA were recruited, as shown in Supplemental Table 1. Venous blood from study volunteers was collected in vacutainer tubes containing spray-coated silica (to prevent red cells from sticking to the tube wall) and a polymer gel for serum separation, then spun at 1,400*g* for 20 minutes. Serum was collected, heat-inactivated at 56°C for 30 minutes, aliquoted, and stored at –80°C. The cellular fraction was resuspended in PBS, and PBMCs were separated from red blood cells using Ficoll extraction and frozen at –80°C before being stored in liquid nitrogen. PBMCs were thawed at 37°C and washed twice before use.

### Anti–SARS-CoV-2 ELISA.

Serologic testing was performed using the FDA-authorized (via Emergency Use Authorization) anti–SARS-CoV-2 IgG ELISA kit from Euroimmun. All testing and analyses were performed according to the manufacturer’s protocols, with the optical density ratio (ODR) calculated using the kit calibrator. The manufacturer-provided reference range is as follows: ratio < 0.8, negative; ratio 0.8 to <1.1, borderline; and ratio ≥ 1.1, positive. To standardize results and facilitate comparisons, ODR scores for each sample were converted to *z* scores (number of SDs above the negative control mean) as follows (44): *z* score = (test ODR – mean negative control ODR)/mean negative control SD. Negative control sera had been collected between 2015 and 2019 from healthy community blood donors and from patients tested in the clinical laboratory by Western blot for potential herpes simplex virus infection (*n* = 78). Based on the negative control data, ODR *z* scores were therefore calculated as (ODR – 0.26)/0.13. A conservative *z* score ≥ 3 was considered positive to minimize false-positive results.

### SARS-CoV-2 RBD protein and tetramer generation.

Recombinant SARS-CoV-2 RBD (from the Wuhan-1 strain, which shares an identical S protein to the WA-1 strain) was generated as previously described (25). For tetramer generation, RBD proteins were biotinylated with the BirA500 kit (Avidity), tetramerized with streptavidin-phycoerythrin (SA-PE) (Agilent, PJRS301-1), and stored in 50% glycerol at –20°C as previously described (45). Decoy reagents were generated by tetramerizing an irrelevant biotinylated protein with SA-PE previously conjugated to Dylight594 NHS Ester (Thermo Fisher Scientific, 46413) and Dylight650 NHS Ester (Thermo Fisher Scientific, 62266).

### SARS-CoV-2 S pseudotyped lentivirus.

The SARS-CoV-2 S pseudotyped lentivirus was produced by transient polyethylenimine transfection of HEK293T cells (ATCC ACS-4500, cultured in DMEM with 10% heat-inactivated FBS, 2 mM l-glutamine, 10 mM HEPES, 100 U/mL penicillin, and 100 μg/mL streptomycin in a humidified atmosphere with 5% CO_2_ at 37°C) with a plasmid encoding the SARS-CoV-2 (WA-1) variant S (D614G mutation and deletion of C-terminal 21aa, BEI Resources NR-53765) or the SARS-CoV-2 (Omicron BA.1, B.1.1.529) variant S (5) and additional components as described (BEI Resources NR-52516, NR-52517, NR-52518, NR-52519). Harvested supernatants were filtered through 0.2 μm filters (Costar, Corning), and viral titers were tested as described (46).

### pVNT.

pVNT assays were performed as previously described (46). Briefly, heat-inactivated plasma was diluted 1:10 followed by four 3-fold serial dilutions all in duplicate and mixed 1:1 with 10^6^ relative luciferase units of SARS-CoV-2 (WA-1) S pseudotyped lentivirus in DMEM with 10% heat-inactivated FBS, 2 mM l-glutamine, 100 U/mL penicillin, and 100 μg/mL streptomycin. After 1-hour incubation at 37°C, the plasma/virus mixtures were added to 96-well, poly-l-lysine–coated plates seeded with human ACE2-expressing HEK293T cells (BEI Resources NR-52511) 20 hours prior. Each plate contained wells with no plasma and HEK293T cells as a background control and a plasma sample from naive individuals (collected early 2020, negative for N- and RBD-specific antibodies) as a negative control (*n* = 4). A monoclonal anti-RBD (WA-1) antibody served as a positive control (10 μg/mL starting dilution), generated by BCR sequencing single-cell-sorted RBD-specific (WA-1) B cells and expressing and purifying the antibody as described (47). After incubating for 48 hours, supernatant was pipetted off and replaced with Bright-Glo Luciferase Assay System luciferase (Promega, E2610) for 2 minutes at 25°C in the dark before transferring to black-bottom plates for measuring luminescence for 1 second per well on a Centro LB 960 Microplate Luminometer (Berthold Technologies). Percentage neutralization was calculated as (1 – [(sample/HEK293T-ACE2 + virus RLU) – (HEK293T + virus RLU)]/[(HEK293T-ACE2 + virus RLU) – (HEK293T + virus RLU)] × 100.

### Immunophenotyping RBD-specific B cells.

PBMCs were thawed at 37°C and washed twice before staining with decoy tetramer and then with RBD tetramer prior to incubation with anti-PE magnetic beads and magnetic bead enrichment (Miltenyi Biotec, 130-048-801) as previously described (45). Cells in the positive fraction were stained with surface antibodies for B cell phenotypes (antibodies listed in Supplemental Table 2).

### Peptide pools.

SARS-CoV-2 15-mer peptides, 1 mg each (BEI Resources), were provided lyophilized and stored at –80°C. Peptides were selected for reactivity against a broad range of class I and class II HLA subtypes for targeted coverage of T cell epitopes as described (5, 48). Before use, peptides were warmed to room temperature for 1 hour, then reconstituted in DMSO to a concentration of 10 mg/mL. Individual peptides were combined in equal ratios to create M/N (182 μg/mL each, 55 peptides) or S (200 μg/mL each, 49 peptides) pools, maintaining a total peptide concentration of 10 mg/mL.

### T cell AIM assay.

For surface phenotyping 10 × 10^6^ PBMCs per sample were divided into four 5 mL polystyrene tubes, and cells were pelleted at 250*g* for 5 minutes. Pellets were resuspended at 5 × 10^6^/mL in one of the following treatment conditions: DMSO (MilliporeSigma, >99.5% cell culture grade), 1 μg/mL CEFX Ultra SuperStim Pool (JPT, PM-CEFX-2), or 5 μg/mL SARS-CoV-2 M/N or S peptide pools. Stimulation was performed for 18 hours in ImmunoCult-XF T Cell Expansion Medium (StemCell Technologies). After stimulation, cells were stained with surface antibodies for T cell activation and phenotype (antibodies listed in Supplemental Table 2).

### Intracellular cytokine assay.

For intracellular cytokine assessment PBMCs (3 × 10^6^/mL) were stimulated using either 10 μg/mL SARS-CoV-2 S protein peptide pool, 50 ng/mL phorbol 12-myristate 13-acetate (MilliporeSigma), and 1 mg/mL ionomycin (MilliporeSigma) or an equivalent volume of DMSO (MilliporeSigma, >99.5% cell culture grade) for 6 hours. This culture occurred in RPMI medium supplemented with FCS, penicillin/streptomycin, sodium pyruvate, and beta-mercaptoethanol. The culture also contained anti-human CD40 antagonist monoclonal antibody (Miltenyi Biotec, clone HB10) to improve resolution of CD154^+^ cells. A total of 1.8 μL monensin (Becton Dickinson) was added for the final 4 hours of culture. Permeabilization and fixation were performed using Cytofix/Cytoperm (Becton Dickinson), and cells were stained with intracellular cytokine antibodies (antibodies listed in Supplemental Table 2).

### Flow cytometry.

Data were acquired on a 5-laser Cytek Biosciences Aurora (T cell surface phenotyping and T cell intracellular cytokine analysis) or BD FACSSymphony A3 or A5 (B cell surface phenotyping). Control PBMCs or UltraComp eBeads (Thermo Fisher Scientific) were used for compensation. Up to 10^7^ live PBMCs were acquired per sample for T cells, and all enriched PBMCs were acquired for B cells. Data were analyzed using SpectroFlow (Cytek Biosciences) and FlowJo 10 (Becton Dickinson) software.

### Statistics.

Statistics are described in figure legends and were determined using Prism (GraphPad). All measurements within a group are from distinct samples. Statistical significance of all pairwise comparisons was assessed by Kruskal-Wallis 1-way ANOVA with Dunn’s post hoc test for multiple comparisons. For correlations, *r*^2^ values are shown to indicate goodness of fit for linear regression, and *P* values are shown to indicate FDR probability of a nonzero slope. Raw *P* values are displayed, and the adjusted *P* value significance cutoff calculated from the Benjamini-Hochberg multiple-testing correction with FDR = 0.05 for each figure is listed in the corresponding legend.

### Study approval.

All samples were obtained upon receipt of written informed consent at the Benaroya Research Institute, part of Virginia Mason Franciscan Health in Seattle, Washington, USA. All studies were approved by the Institutional Review Board of the Benaroya Research Institute.

### Data availability.

All raw data presented in this paper are available in the Supporting Data Values spreadsheet.

## Author contributions

SDK, LBR, YY, EB, JHB, CS, MP, and DJC conceptualized and designed the study. CS and JHB supervised patient selection and recruitment efforts. SDK and LBR performed most experiments. CM and MHW developed and performed the anti-S protein–specific ELISAs. SDK, LBR, and DJC performed data analysis and interpretation and wrote the manuscript with assistance from all coauthors. DJC and JHB obtained funding, and DJC supervised the study.

## Supplementary Material

Supplemental data

Supporting data values

## Version 1 06/20/2023

In-Press Preview

## Version 2 08/08/2023

Electronic publication
