# Supplementary material for: Diminished responses to mRNA-based SARS-CoV-2 vaccines in individuals with rheumatoid arthritis on immune-modifying therapies
Source: JCI Insight. 2023 Aug 8;8(15):e168663. doi: 10.1172/jci.insight.168663 (PMC10445680; doi:10.1172/jci.insight.168663)
Supplement: Supplemental data [file jciinsight-8-168663-s106.pdf]

Table S1

Subject ID	Subject Type	Active Autoimmune Medications	Days between Vaccine Dose 2 and Blood Draw	Vaccine type	Age at Draw	Race	Gender	Rheumatoid Arthritis: Duration At Draw (years)	Disease classification (anti-CCP & rheumatoid factor antibody results)	RA Erosion Xray	Aproximate time between last dose of rituximab or abatacept and Vaccine Dose 1 (days)
Control_01	Healthy Control		15	mRNA-1273	37	Decline (Patient Refused)	female				
Control_02	Healthy Control		17	mRNA-1273	37	White, Caucasian	male				
Control_03	Healthy Control		7	BNT162b1 and BNT162b2 mRNA-based vaccine	40	Asian, White, Caucasian	female				
Control_04	Healthy Control		13	BNT162b1 and BNT162b2 mRNA-based vaccine	50	White, Caucasian	male				
Control_05	Healthy Control		14	BNT162b1 and BNT162b2 mRNA-based vaccine	50	White, Caucasian	female				
Control_06	Healthy Control		7	mRNA-1273	51	White, Caucasian	female				
Control_07	Healthy Control		10	BNT162b1 and BNT162b2 mRNA-based vaccine	52	White, Caucasian	male				
Control_08	Healthy Control		19	BNT162b1 and BNT162b2 mRNA-based vaccine	53	White, Caucasian	male				
Control_09	Healthy Control		14	BNT162b1 and BNT162b2 mRNA-based vaccine	55	White, Caucasian	male				
Control_10	Healthy Control		11	mRNA-1273	56	White, Caucasian	male				
Control_11	Healthy Control		18	mRNA-1273	57	White, Caucasian	female				
Control_12	Healthy Control		12	mRNA-1273	60	Asian	female				
Control_13	Healthy Control		13	mRNA-1273	68	White, Caucasian	female				
Abatacept_01	RA	Abatacept	14	BNT162b1 and BNT162b2 mRNA-based vaccin	37	Asian	female	9.7	CCP+, RF+	Non-erosive	28
Abatacept_02	RA	Abatacept	35	mRNA-1273	48	White, Caucasian	female	18.4	CCP+, RF+	Non-erosive	<30
Abatacept_03	RA	Abatacept, Hydroxychloroquine, Prednisone	13	BNT162b1 and BNT162b2 mRNA-based vaccine	50	Asian	female	15.4	RF+, CCP+	Non-erosive	<7
Abatacept_04	RA	Abatacept, Hydroxychloroquine, Prednisone	15	BNT162b1 and BNT162b2 mRNA-based vaccine	51	White, Caucasian	female	28.1	CCP+, RF+	Not available	<30
Abatacept_05	RA	Abatacept, Hydroxychloroquine	20	mRNA-1273	58	Asian, White, Caucasian	female	20.7	CCP+, RF+	Not available	<30
Abatacept_06	RA	Abatacept, Methotrexate	12	BNT162b1 and BNT162b2 mRNA-based vaccin	58	White, Caucasian	female	11	CCP-	Not available	<30
Abatacept_07	RA	Abatacept, Sulfasalazine	94	mRNA-1273	62	White, Caucasian	female	7	CCP-, RF-	Non-erosive	<7
Abatacept_08	RA	Abatacept	21	BNT162b1 and BNT162b2 mRNA-based vaccin	65	Asian	male	7	CCP+	Not available	<7
Abatacept_09	RA	Abatacept, Hydroxychloroquine, Leflunomide	22	mRNA-1273	67	Decline (Patient Refused)	female	17.4	CCP-, RF-	Non-erosive	<30
Abatacept_10	RA	Abatacept	88	BNT162b1 and BNT162b2 mRNA-based vaccin	69	White, Caucasian	female	27.6	CCP+, RF+	Erosive	<30
Abatacept_11	RA	Abatacept	78	BNT162b1 and BNT162b2 mRNA-based vaccine	76	American Indian, Alaska Native	female	6.8	RF+	Non-erosive	<7
DMARD_01	RA	Methotrexate	25	mRNA-1273	42	White, Caucasian	male	3.1	CCP-, RF-	Not available	NA
DMARD_02	RA	Methotrexate	47	BNT162b1 and BNT162b2 mRNA-based vaccin	44	White, Caucasian	female	1.9	CCP+, RF+	Non-erosive	NA
DMARD_03	RA	Methotrexate	9	BNT162b1 and BNT162b2 mRNA-based vaccin	48	White, Caucasian	female	JIA In Remission	CCP-	Not available	NA
DMARD_04	RA	Methotrexate	10	BNT162b1 and BNT162b2 mRNA-based vaccin	49	White, Caucasian	female	22	CCP+, RF+	Non-erosive	NA
DMARD_05	RA	Methotrexate	15	BNT162b1 and BNT162b2 mRNA-based vaccin	55	White, Caucasian	male	1.4	CCP+, RF-	Erosive	NA
DMARD_06	RA	Methotrexate, Prednisone	11	BNT162b1 and BNT162b2 mRNA-based vaccin	55	White, Caucasian	female	21.1	CCP+, RF-	Erosive	NA
DMARD_07	RA	Methotrexate	98	BNT162b1 and BNT162b2 mRNA-based vaccin	62	White, Caucasian	female	3.5	CCP-, RF-	Not available	NA
DMARD_08	RA	Methotrexate (held during vaccination	13	BNT162b1 and BNT162b2 mRNA-based vaccin	63	White, Caucasian	female	0.9	CCP-	Not available	NA
DMARD_09	RA	Methotrexate	14	mRNA-1273	64	Native Hawaiian, Other Pacific Islander	female	3.1	CCP-	Not available	NA
DMARD_10	RA	Methotrexate	170	mRNA-1273	70	White, Caucasian	male	6	CCP+	Not available	NA
DMARD_11	RA	Methotrexate	126	mRNA-1273	74	Asian	female	16	CCP-	Not available	NA
Rituximab_1	RA	Rituximab, Hydroxychloroquine	23	mRNA-1273	54	American Indian, Alaska Native, White, Caucasian	female	20	CCP-	Not available	162
Rituximab_2	RA	Rituximab	21	mRNA-1273	55	White, Caucasian	female	28	CCP+	Not available	160
Rituximab_3	RA	Rituximab, Hydroxychloroquine, Methotrexate	36	BNT162b1 and BNT162b2 mRNA-based vaccine	56	Asian	female	22.9	CCP+, RF+	Not available	161
Rituximab_4	RA	Rituximab	14	mRNA-1273	64	White, Caucasian	female	4.3	CCP+, RF-	Erosive	199
Rituximab_5	RA	Rituximab, Leflunomide, Methotrexate	9	BNT162b1 and BNT162b2 mRNA-based vaccine	72	White, Caucasian	female	17.2	RF+	Not available	39

Supplemental Table 1.
Demographic and disease information for all subjects in the study.

Table S2

Panel	Antibody	Clone	Supplier
B cell panel	Live/Dead fixable blue		Thermo Scientific
	CD3 :: PerCP/Cy5.5	HIT3a	BioLegend
	CD14 :: PerCP/Cy5.5	M5E2	BioLegend
	CD16 :: PerCP/Cy5.5	3G8	BioLegend
	CD19 :: BUV496	SJ25C1	BD Biosciences
	CD20 :: BV711	2H7	BioLegend
	CD38 :: Alexa700	90	ThermoFisher
	CD21 :: SB600	HB5	ThermoFisher
	CD27 :: BV421	M-T271	BioLegend
	IgD :: BUV395	IA6-2	BD Biosciences
	IgM :: BV510	MHM-88	BioLegend
	IgG :: BV786	G18-145	BD Biosciences
	IgA :: PE/Vio770	IS11-8E10	Miltenyi Biotec
	CD11c :: PE/Dazz594	3.9	BioLegenc
T cell AIM panel	CD45 :: BUV395	HI30	BD Biosciences
	CD45 :: BUV496	HI30	BD Biosciences
	CD45 :: efluor450	HI30	ThermoFisher
	CD45 :: Alexa532	HI30	ThermoFisher
	CD3 :: BUV615	UCHT1	BD Biosciences
	HLA-DR :: BUV661	G46-6	BD Biosciences
	CD45RA :: BUV737	HI100	BD Biosciences
	CD26 :: BUV805	M-A261	Thermo Scientific
	CXCR3 :: BV421	1C6	BD Biosciences
	CD8a :: BV480	RPA-T8	BD Biosciences
	CCR7 :: BV605	G043H7	BioLegend
	CCR6 :: BV650	G043G3	BioLegend
	CD27 :: BV711	M-T271	BioLegend
	CD137 :: BV750	4B4-1	BioLegend
	CD57 :: BV785	QA17A04	BioLegend
	CXCR5 :: BB515	RF8B2	BD Biosciences
	CD134 :: PerCP/Cy5.5	BerACT35	BioLegend
	PDL1 :: PE	29E.2A3	BioLegend
	CCR4 :: PE/Dazz594	L291H4	BioLegend
	CD25 :: PE/Cy5	BC96	BioLegend
	CD127 :: PE/Cy7	hIL7Rm21	BioLegend
	ICOS :: APC	C398.4a	BioLegend
	CD4 :: Spark685 NIR	SK3	BioLegend
	CD69 :: APC/R700	FN50	BD Biosciences
	Live/Dead Zombie NIR		BioLegend
	gdTCR :: APC/Fire750	B1	BioLegend
	CD19 :: APC/Fire810	HIB19	BioLegenc
T cell ICS panel	CD69 :: BUV395	FN50	BD Biosciences
	Live/Dead fixable blue		eBioscience
	IL-13 :: BV421	JES10-5E2	BioLegend
	CD3 :: efluor450	OKT3	eBioscience
	CD107a :: BV510	H4A3	BioLegend
	IL-17A :: BV570	BL168	BioLegend
	CD40L :: Biotin	hCD40L-M91	BD Biosciences
	Streptavidin :: BV605	563260	BD Biosciences
	CD25 :: BV650	M-A251	BD Biosciences
	CD19 :: BV711	SJ25C1	BD Biosciences
	CD16 :: BV711	3G8	BD Biosciences
	CD14 :: BV711	MOP9	BD Biosciences
	CD45RA :: BV711	HI100	BD Biosciences
	IL-2 :: BV785	MQ1-17H12	BioLegend
	CD127 :: Alexa488	AO19D5	BioLegend
	IL-21 :: PE	3A3-N2	eBioscience
	IL-10 :: PE/Dazz594	JES3-9D7	BioLegend
	CD8 :: PE/Cy5	RPA-T8	BD Biosciences
	IL-4 :: PE/Cy7	MP4-25D2	BioLegend
	CXCR5 :: Alexa647	J252D4	BioLegend
	CD4 :: Alexa700	RPA-T4	BD Biosciences
	IFNy :: APC/efluor780	4S-B3	eBioscience

Supplemental Table 2.
List of antibodies (marker/fluorophore) used in each flow cytometry panel, with antibody clone and supplying company indicated.

Figure S1

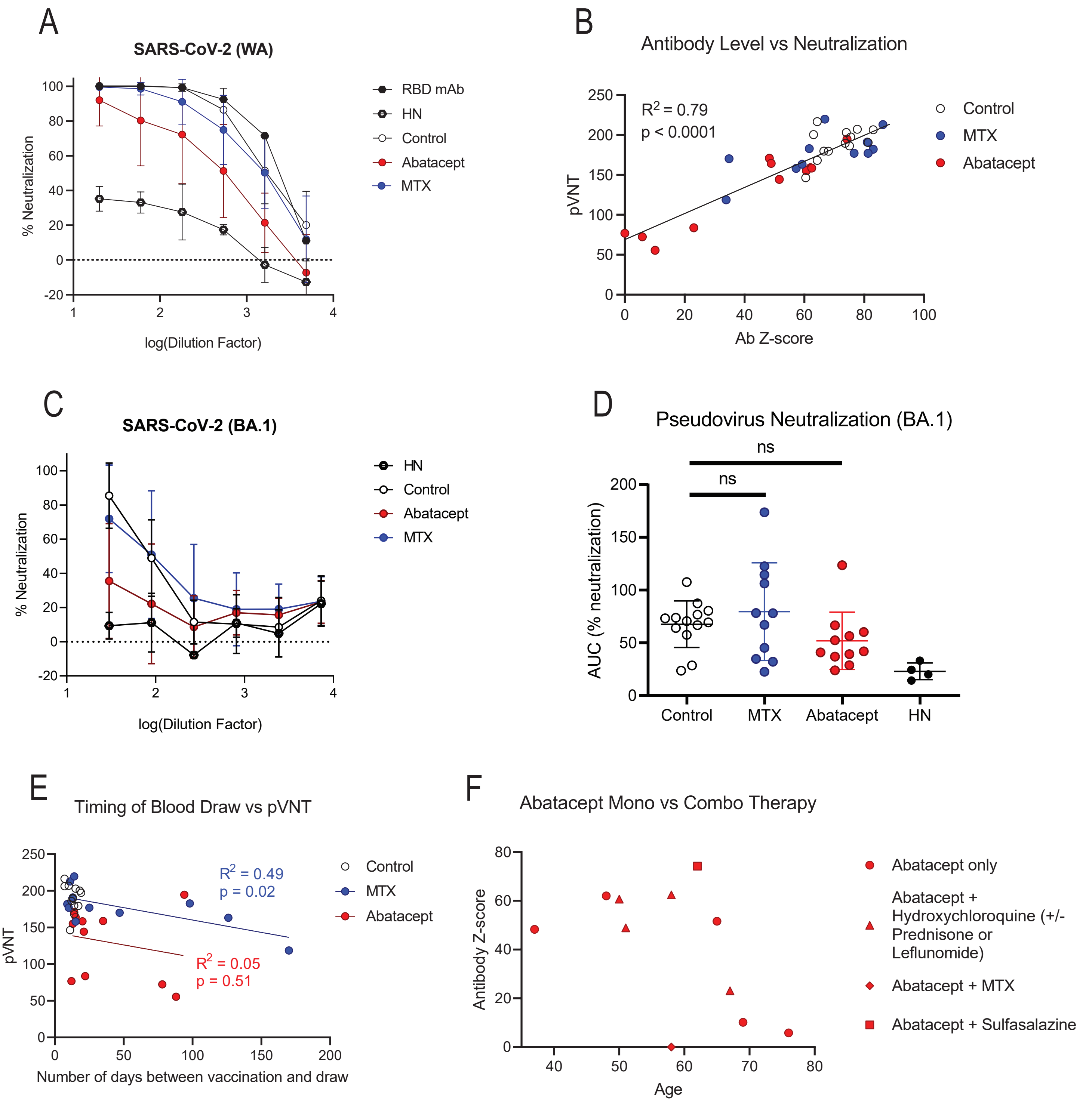

Supplemental Figure 1.
(A) Percent neutralization of WA-1 S-pseudotyped lentivirus infection of ACE2-expressing cells across all serum dilutions tested, with anti-RBD monoclonal antibody (mAb) as positive control and historical/naive (HN) as negative control. **(B)** Anti-S antibody levels graphed against pseudovirus neutralization. **(C)** Percent neutralization of Omicron BA.1 S-pseudotyped lentivirus infection of ACE2-expressing cells across all serum dilutions tested, with historical/naive (HN) as negative control. **(D)** Neutralization of subjects' sera against BA.1-typed pseudovirus, as area-under-curve (AUC) across serum dilutions. **(E)** Time between each subject's second vaccine dose and blood draw for the study graphed against pseudovirus neutralization. **(F)** Age graphed against antibody Z score for abatacept subjects, split by individuals on mono vs combination therapy. *Error bars represent mean +/- SD. All linear regression shown with r-squared values and p values testing probability of a non-zero slope.*

Figure S2

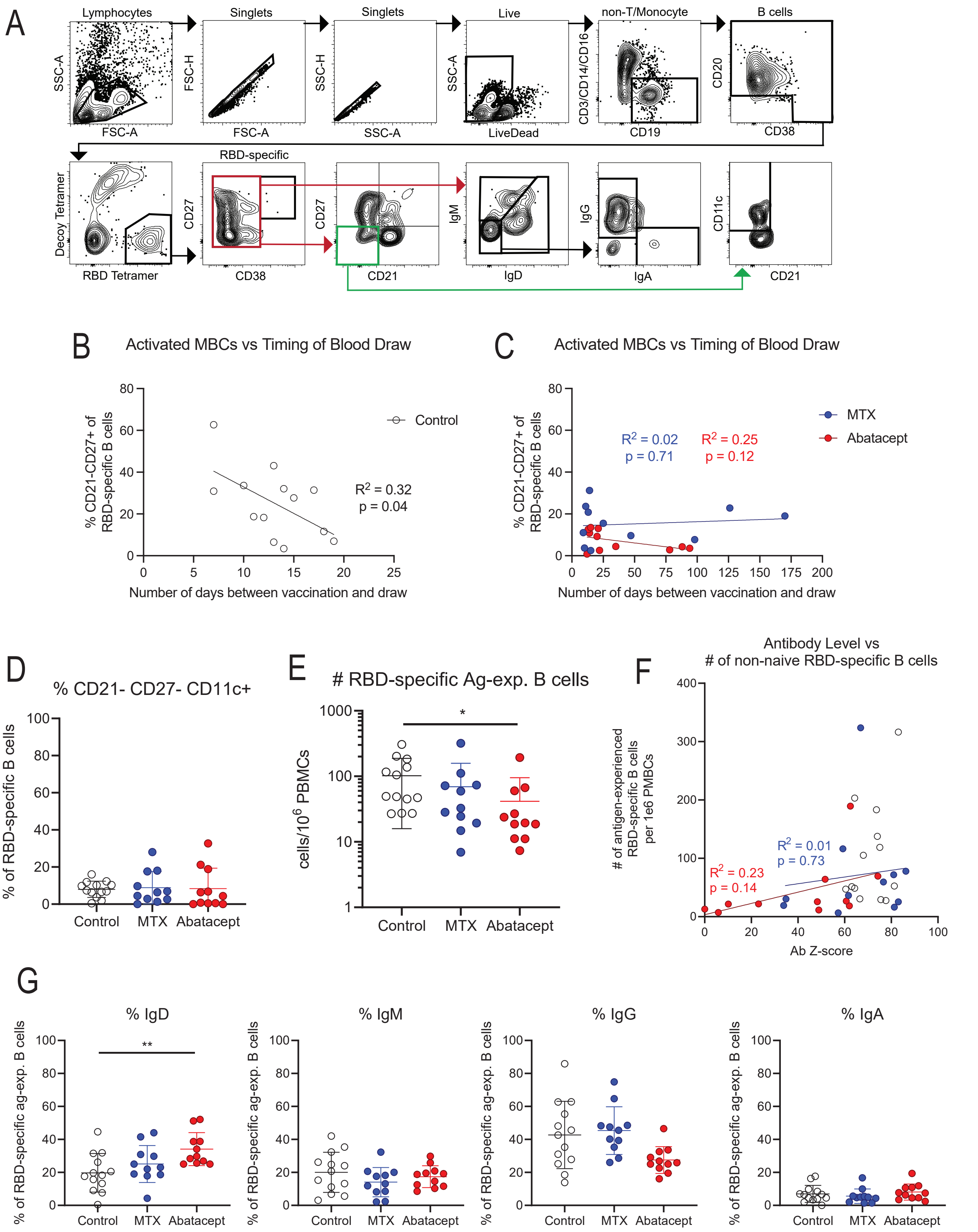

Supplemental Figure 2.
(A) Gating strategy for identifying and phenotyping RBD-specific B cells from PBMCs. **(B,C)** Correlation between time since second vaccine dose and percent activated ($CD21^-CD27^+$) MBCs for control (B) and RA (C) groups. **(D)** Percent of atypical MBCs ($CD21^-CD27^-CD11c^+$) of RBD-specific B cells. **(E)** Number of antigen-experienced (Ag-exp., $CD21^+CD27^+$ or $CD21^-CD27^{+/-}$) RBD-specific B cells and **(F)** correlation with normalized quantity of S-specific antibody. Linear regression lines for abatacept and MTX groups. **(G)** Percent of RBD-specific antigen-experienced B cells expressing each isotype indicated. *Error bars represent mean +/- SD. All linear regression shown with r-squared values and p values testing probability of a non-zero slope. Statistics determined by Kruskal-Wallis test with post-hoc Dunn's multiple comparison test. All statistically significant comparisons (p<.05) are shown. * p<.05, ** p<.01, *** p<.001*

Figure S3

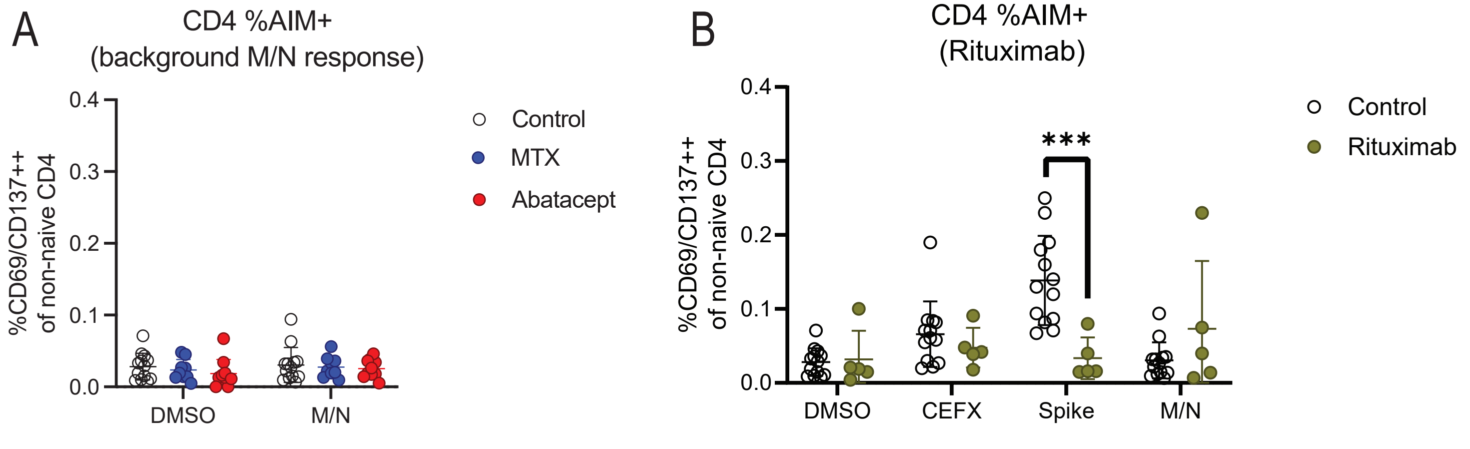

Supplemental Figure 3.
(A) Percent AIM+ data from T cell stimulation assay shown for the membrane/nucleocapsid control condition.
(B) Percent AIM+ data from T cell stimulation assay shown for the RA cohort on rituximab. *Error bars represent mean +/- SD. Statistics determined by Mann-Whitney test. *** p<.001*

Figure S4

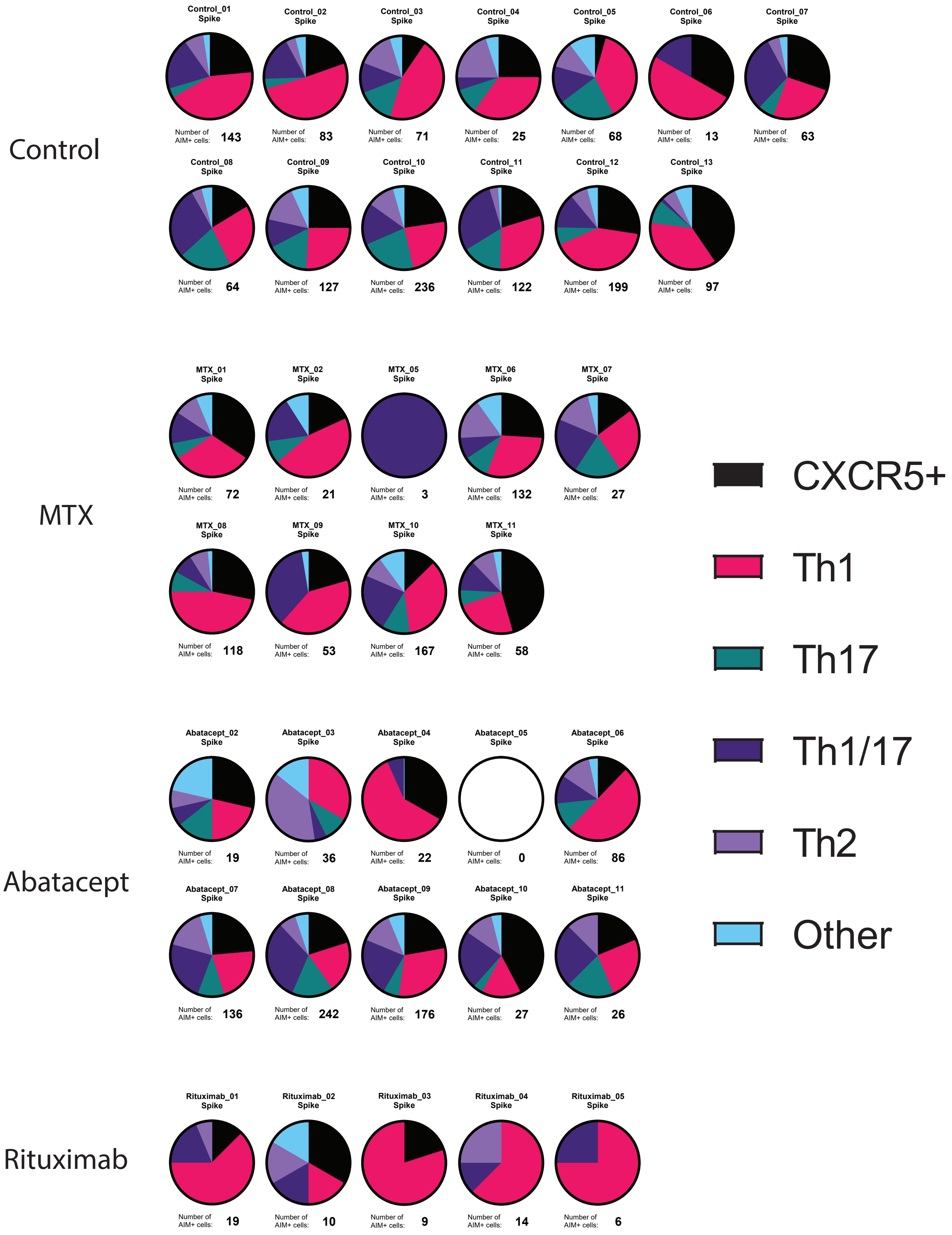

Supplemental Figure 4.
Pie charts showing percentage of AIM+ CD4 T cells falling into each Th subset shown for every individual donor, with the number of AIM+ CD4s in each donor indicated.

Figure S5

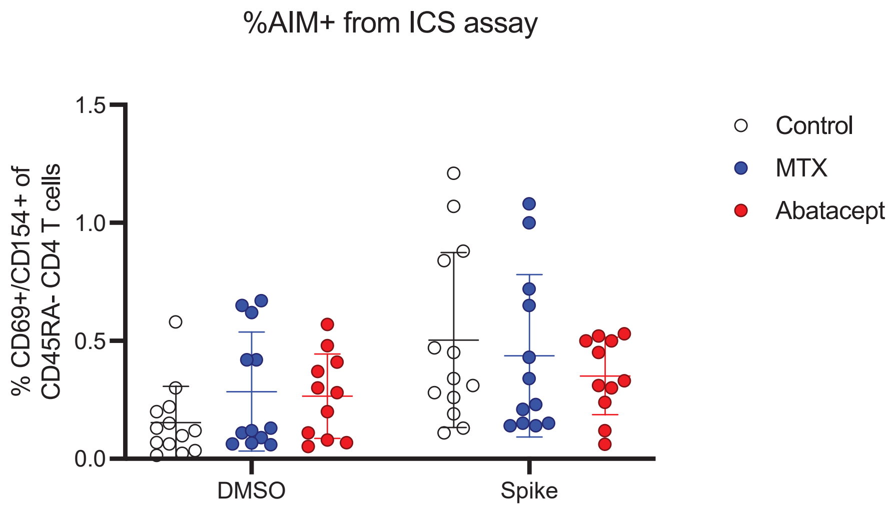

Supplemental Figure 5.
Percentage of AIM+ ($CD69^{+}CD154^{+}$) non-naïve CD4 T cells after DMSO or Spike stimulation in ICS assay co-culture. *Error bars represent mean +/- SD. Kruskal-Wallis test with post-hoc Dunn's multiple comparison test showed no significant (p<.05) differences.*
